# Antitrypanosomal Activities and Mechanisms of Action of Novel Tetracyclic Iridoids from Morinda lucida Benth.

**DOI:** 10.1128/AAC.01916-15

**Published:** 2016-05-23

**Authors:** Kofi D. Kwofie, Nguyen Huu Tung, Mitsuko Suzuki-Ohashi, Michael Amoa-Bosompem, Richard Adegle, Maxwell M. Sakyiamah, Frederick Ayertey, Kofi Baffour-Awuah Owusu, Isaac Tuffour, Philip Atchoglo, Kwadwo K. Frempong, William K. Anyan, Takuhiro Uto, Osamu Morinaga, Taizo Yamashita, Frederic Aboagye, Alfred A. Appiah, Regina Appiah-Opong, Alexander K. Nyarko, Yasuchika Yamaguchi, Dominic Edoh, Kwadwo A. Koram, Shoji Yamaoka, Daniel A. Boakye, Nobuo Ohta, Yukihiro Shoyama, Irene Ayi

**Affiliations:** a Noguchi Memorial Institute for Medical Research, College of Health Sciences, University of Ghana, Legon, Ghana; b Section of Environmental Parasitology, Faculty of Medicine, Tokyo Medical and Dental University, Bunkyo-ku, Tokyo, Japan; c Faculty of Pharmaceutical Sciences, Nagasaki International University, Sasebo, Nagasaki, Japan; d Centre for Scientific Research into Plant Medicine, Mampong-Akuapem, Ghana

## Abstract

Trypanosoma brucei parasites are kinetoplastid protozoa that devastate the health and economic well-being of millions of people in Africa through the disease human African trypanosomiasis (HAT). New chemotherapy has been eagerly awaited due to severe side effects and the drug resistance issues plaguing current drugs. Recently, there has been an emphasis on the use of medicinal plants worldwide. Morinda lucida Benth. is a popular medicinal plant widely distributed in Africa, and several research groups have reported on the antiprotozoal activities of this plant. In this study, we identified three novel tetracyclic iridoids, molucidin, ML-2-3, and ML-F52, from the CHCl_3_ fraction of M. lucida leaves, which possess activity against the GUTat 3.1 strain of T. brucei brucei. The 50% inhibitory concentrations (IC_50_) of molucidin, ML-2-3, and ML-F52 were 1.27 μM, 3.75 μM, and 0.43 μM, respectively. ML-2-3 and ML-F52 suppressed the expression of paraflagellum rod protein subunit 2, PFR-2, and caused cell cycle alteration, which preceded apoptosis induction in the bloodstream form of Trypanosoma parasites. Novel tetracyclic iridoids may be promising lead compounds for the development of new chemotherapies for African trypanosomal infections in humans and animals.

## INTRODUCTION

Human African trypanosomiasis (HAT), commonly known as sleeping sickness, has remained a serious health problem in many African countries with thousands of new cases of infection annually (1, 2). Although millions of people are under threat of HAT in Africa, it is known as one of the neglected diseases for which there is a lack of the necessary resources to bring new compounds to market for possible drug development (3, 4). HAT is caused by protozoan parasites belonging to the genus Trypanosoma and is transmitted through the bites of tsetse flies. In Africa, there are mainly two species responsible for the disease; T. brucei gambiense and T. brucei rhodesiense. T. brucei gambiense is responsible for about 98% of reported cases of sleeping sickness while T. brucei rhodesiense is responsible for 2% of reported cases (2). In 2012, 7,216 cases were reported with emphasis on the complexity of diagnosis; therefore, skilled personnel for case detection will be needed (2).

The current treatments for HAT are far from ideal (5). Chemotherapeutic agents against HAT, namely, suramin, pentamidine, melarsoprol, and eflornithine (3, 6–8), cause severe side effects (9), require lengthy parenteral administration, and are unaffordable for most patients. In addition to those concerns, the increase in drug resistance urges the need for the discovery of new chemotherapeutic agents against HAT (10, 11).

Recently, there has been emphasis on the use of medicinal plants worldwide (12–14). Morinda lucida Benth. (Rubiaceae), an evergreen medium-sized tree with dark-shiny leaves on the upper surface, is one of the most popular medicinal plants widely distributed in Africa (15). Phytochemical studies showed that M. lucida is a natural resource rich in anthraquinones like oruwacin, oruwal, 3-hydroxyanthraquinone-2-carboxyaldehyde, 1,3-dihydroxy-2-methylanthraquinone, 1,3-dihydroxyanthraquinone-2-carboxyaldehyde, and many others (16–19). It is used among traditional healers to treat fever, dysentery, abdominal colic, and intestinal worm infestation. Several groups have reported on the antiprotozoal activities of M. lucida, and some active compounds isolated have been from it (20–25). Anthraquinones isolated from M. lucida are reported to have antileishmanial and antimalarial activities (26). Three other compounds purified from M. lucida were also reported to have high activities against Plasmodium falciparum (20, 21). Although several groups have revealed the antitrypanosomal activities of M. lucida crude extracts, the responsible compounds have not yet been isolated (27, 28).

We previously reported on the antitrypanosomal activity of the novel tetracyclic iridoid molucidin (29). In the present study, we report, in addition to molucidin, the antitrypanosomal activities of two more novel tetracyclic iridoid**s,** namely, ML-2-3 and ML-F52**,** as well as 3 other known compounds **(**oruwalol, ursolic acid [30], and oleanolic acid) isolated from the leaves of M. lucida. In this study, we also report on the role of these compounds in apoptosis induction and cell cycle alteration in trypanosome parasites. It is also known that the Trypanosoma flagellum plays a key role not only in motility but also in morphology, growth, and cell division. In the kinetoplastid flagellum, there is a major protein known as the paraflagellar rod (PFR), which runs adjacent to the canonical 9 + 2 axoneme structure. The paraflagellar rod consists of 2 protein subunits referred to as PFR-1 and PFR-2 (31–33). The important role of the PFR-2 protein in flagellum function was demonstrated when parasite mutants lacking the PFR-2 protein exhibited reduced swimming velocity and paralyzed phenotype and, hence, a reduction in survival rates (34, 35). The PFR-2 protein appears to be a potential choice of target for the development of new chemotherapy. In this study, we therefore report on the effect of the compounds on the expression of the PFR-2 protein and parasite morphology. Activity and mechanistic results with novel tetracyclic iridoids, molucidin, ML-2-3, and ML-F52, suggest that they are promising lead compounds for the development of new drugs against the kinetoplastid protozoan, Trypanosoma brucei.

## MATERIALS AND METHODS

### Plant material and general procedures.

This study involved the screening of several extracts from different parts of about 73 Ghanaian medicinal plants that were selected according to traditional knowledge for antitrypanosomal activity. Morinda lucida was found to have the strongest antitrypanosomal activity among them.

The leaves of M. lucida were collected in Mampong, Ghana in 2012 and were authenticated by one of the authors (Y. Shoyama). Voucher specimens have been deposited at the Department of Pharmacognosy, Nagasaki International University, Japan, and at the Centre for Scientific Research into Plant Medicine, Ghana. Plant materials (crude extracts) were screened *in vitro* against trypanosomes for trypanocidal activity. Extracts with activity were fractionated, and the resulting fractions were screened in the same manner. Fractions found to have antitrypanosomal activity were further processed to isolate compounds that were likewise screened for activity. Compounds with high activities were selected to establish their mechanism of action, and their structures were elucidated. An established 3-step screening system described elsewhere in the manuscript was employed. Optical rotations were obtained using a DIP-360 digital polarimeter (Jasco, Easton, MD, USA). Nuclear magnetic resonance (NMR) spectra were recorded on a JEOL ECX 400 NMR spectrometer (Tokyo, Japan). High-resolution electrospray ionization time-of-flight mass spectrometry (HR-ESI-TOFMS) experiments utilized a JEOL AccuTOF LC 1100 mass spectrometer (Tokyo, Japan). Column chromatography was performed on silica gel 60 (230–400 mesh; Nacalai Tesque Inc., Kyoto, Japan) and YMC ODS-A gel (50 μm; YMC Co. Ltd., Kyoto, Japan). Thin-layer chromatography (TLC) was performed on Kieselgel 60 F_254_ (Merck, Damstadt, Germany) plates. Spots were visualized by spraying with 10% aqueous H_2_SO_4_ solution followed by heating.

### Isolation of compounds.

Air-dried and pulverized leaf samples of Morinda lucida (1,100 g) were extracted with 50% aqueous ethyl alcohol (EtOH) (2.0 liters 3 times) at 40°C under sonication. After removal of solvent, the obtained residue (203 g) was suspended in 1.0 liter of water and successively partitioned with (1.0 liter × 3 each) hexane, CHCl_3_, and ethyl acetate (EtOAc) to obtain soluble fractions of hexane (2.1 g), CHCl_3_ (3.80 g), and EtOAc (3.6 g). The CHCl_3_ fraction, the most active fraction against Trypanosoma, was subjected to a silica gel column (45 by 350 mm) fractionation with hexane-EtOAc (2:1, vol/vol) as the mobile phase to give seven subfractions (fr.1∼fr.7). fr.1 (120 mg) was then rechromatographed over a reversed-phase (RP) column (20 by 450 mm) with methanol (MeOH)-H_2_O (10:1, vol/vol) to yield compounds 4 (white powder, 15 mg) and 5 (white powder, 18 mg). fr.2 (80 mg) was further chromatographed over a RP column (20 by 450 mm) with MeOH-H_2_O (1:1, vol/vol) to obtain compound 1 (yellow solid, 30 mg). Similarly, fr.4 (140 mg) was loaded onto a RP column (20 by 450 mm) with MeOH-H_2_O (3:2, vol/vol) to yield compound 3 (colorless crystal, 35 mg). Subsequently, compound 2 (colorless crystal, 50 mg) was purified from fr.6 (550 mg) by means of a RP column (30 by 400 mm) with MeOH-H_2_O (3:5, vol/vol) followed by a silica gel column (20 by 350 mm) with CHCl_3_-MeOH (25:1, vol/vol).

### Screening of compounds for antikinetoplastid activities. (i) Trypanosome parasites.

The GUTat 3.1 strain of the bloodstream form of T. brucei brucei parasites was used in this study. Parasites were cultured *in vitro* according to the conditions established previously (36). Parasites were used when they reached a confluent concentration of 1 × 10^6^ parasites/ml. Estimation of parasitemia was done with the Neubauer counting chamber. Parasites were diluted to a concentration of 3 × 10^5^ parasites/ml with HM1-9 medium and were used for the various experiments.

### (ii) *In vitro* viability test for trypanosome parasites.

The alamarBlue assay (Life Technologies, USA) was carried out on treated or untreated trypanosome parasites to ascertain their viability. The assay was performed in a 96-well plate following the manufacturer's instructions with modification. Briefly, 1.5 × 10^4^ parasites were seeded with varied concentrations of plant material (extracts, fractions, or compounds) ranging from 0.78 μg/ml to 200 μg. Final concentrations of EtOH and dimethyl sulfoxide (DMSO) were kept at <1% and 0.1%, respectively. After incubation of parasites with or without plant extracts or compounds for 24 h at 37°C in 5% CO_2_, 10% alamarBlue dye was added, and the parasites were incubated another 24 h in darkness. After a total of 48 h, the plate was read for absorbance at 540 nm using the Tecan Sunrise Wako spectrophotometer. The trend curve was drawn to obtain a 50% inhibitory concentration (IC_50_) of each plant material (extracts, fractions, and compounds).

### Testing of compounds for cytotoxicity to mammalian cells. (i) Cell cultures for cytotoxicity assay.

The cytotoxicity of the compounds to mammalian cells were determined using four human normal cell lines, namely, NB1RGB (skin fibroblast) and HF-19 (lung fibroblast) obtained from the RIKEN Bio Resource Center Cell Bank (Japan) and Chang Liver and Hs888Lu (lung) obtained from the European Collection of Authenticated Cell Cultures (ECACC). NB1RGB and HF-19 were maintained in minimum essential medium-α (MEM-α). Chang Liver and Hs888Lu were grown in Eagle's minimum essential medium (EMEM) and RPMI 1640, respectively. All of these media were supplemented with 10% fetal bovine serum (FBS) and 1% penicillin-streptomycin and were then incubated at 37°C under 5% CO_2_ in fully humidified conditions.

Cytotoxicity was determined using a 3-(4,5-dimethylthiazol-2-yl)-2,5-diphenyltetrazolium bromide (MTT) assay. The cells were treated with molucidin, ML-2-3, or ML-F52 at concentrations of ≤50 μM for 48 h. Cells were plated at a density of 0.5 × 10^4^ cells/well into 96-well plates. After 24 h of incubation, cells were treated with various concentrations of each of the purified compounds for 48 h. Then, MTT solution was added to each well, and the cells were incubated for another 4 h. The precipitated MTT-formazan product was dissolved in 0.04 N HCl-isopropanol, and the amount of formazan was measured at a wavelength of 595 nm by a microplate reader (Immuno Mini NJ-2300; Nihon InterMed, Tokyo, Japan). Cytotoxicity was calculated as the percentage of live cells relative to the control culture. The selectivity index (SI) was expressed as the ratio of the IC_50_ obtained for mammalian cells and the IC_50_ for trypanosomes.

### (ii) Fluorescence-activated cell sorter analysis for detection of apoptosis and cell cycle alteration.

Trypanosoma cells were treated with either 6.25 μM molucidin (about 5 times that of the IC_50_), 6.25 μM ML-2-3 (about 2 times that of the IC_50_), or 0.78 μM ML-F52 (about 2 times that of the IC_50_) for 24 h and were then subjected to the Nexin assay.

Seeding and incubation of parasites with compounds were done under the same conditions as those for the alamarBlue assay as described above without addition of the alarmarBlue reagent. In this case, after 24 h of incubation, Guava reagents for Nexin and cell cycle assays were added, and each assay was performed using the Millipore Guava easyCyte 5HT fluorescence-activated cell sorter (FACS) machine according to the manufacturer's instruction. The Nexin assay and subsequent FACS analysis allowed for detection of markers of apoptosis induction by the plant materials. Similarly, the cell cycle assay and subsequent FACS analysis allowed for detection of markers of cell cycle alteration by the plant materials.

### Investigating the effect of compounds on parasite morphology and flagella function.

To investigate the effect of the compounds on parasite morphology and their flagellum function, immunohistochemistry using anti-paraflagellum rod protein anti-PFR-2 antibody (37), was performed with molucidin-, ML-2-3-, and ML-F52-treated trypanosome parasites. Briefly, parasites were incubated for 24 h under appropriate conditions (37°C, 5% CO_2_) with 5 μM (4 times that of the IC_50_) of molucidin, 15 μM (4 times that of the IC_50_) of ML-2-3, and 0.43 μM (IC_50_) of ML-F52. Parasites were then harvested after incubation with or without appropriate concentrations of compounds and were fixed with 4% paraformaldehyde in 8-well chamber slides at room temperature for 5 min. Washing steps were carried out with 500 μl of phosphate-buffered saline (PBS) twice and 0.1% Triton X-100 in PBS (PBST) at room temperature for 5 min each. Blocking reagent (500 μl; 3% bovine serum albumin [BSA] in PBS) was added and incubated for 30 min at room temperature. Primary and secondary antibody incubation with parasites was done for 1 h each, and 4′,6-diamidino-2-phenylindole (DAPI) (5 μg/ml DAPI in PBS) staining was done for 10 min. After the washing steps presented above, the slides were mounted using PermaFluor mounting reagent and were covered with coverslips. The slides were observed under an Olympus fluorescence microscope (Olympus BX53) to detect any phenotypic changes in T. brucei brucei parasites.

### PFR-2 protein expression analysis.

Trypanosome parasites were incubated with or without compound *in vitro* and were lysed with 0.5% NP-40 (38). Protein concentration of the lysate was determined using the Bio-Rad protein assay reagents (Bio-Rad, USA). SDS-PAGE was run using Invitrogen NuPAGE 12% Bis-Tris gel. The proteins were blotted on a polyvinylidene difluoride (PVDF) membrane (Immobilon P; Millipore, USA), added with mouse anti-PFR-2 antibody, 1:500 dilution, and incubated at 4°C overnight. The membrane was then incubated with anti-mouse horseradish peroxidase (HRP) antibodies, 1:2,000 dilution, for an hour at room temperature. Chemiluminescence HRP substrates A and B (Immobilon Western; Millipore, USA) were added to the membrane in a ratio of 1:1. Detection was done using the ATTO cooled charge-coupled device (CCD) camera system EzCapture II (ATTO Corporation, Japan).

### Time course analysis for mechanisms of action by compounds.

To investigate the sequence in which the events (apoptosis induction, PFR-2 suppression, and cell cycle alteration) involved in mechanisms of action occur, we examined the time course for the three events using ML-2-3-treated parasites. Trypanosoma brucei parasites were incubated for 0, 0.5, 1.5, 3, 6, and 24 h with 15 μM ML-2-3 and were then subjected to both Nexin assay and Western blot analyses using the PFR-2 antibody. We further investigated the time course of cell cycle alteration using ML-2-3-treated parasites at similar concentrations and incubation periods.

### Structural analysis and comparison of compounds.

In addition to the comparison of its spectroscopic data with those of plumericin, prismatomerin, and oruwacin, the relative configuration of ML-2-3 was then elucidated by an nuclear Overhauser effect spectroscopy (NOESY) experiment.

### *In vivo* efficacy assay for active compounds.

Six-week-old BALB/c female mice with an average weight of 20 g were infected with 1 × 10^3^ cells of T. brucei brucei (TC-221 strain) and were randomly grouped into four cages containing five mice each. The first 3 groups were treated with 30 mg/kg of body weight of molucidin, ML-2-3, and ML-F52, respectively, 6 h postinfection and continued daily afterward for 5 consecutive days. The last group received physiological saline containing <0.1% of DMSO as a vehicle control. Parasitemia and weight were monitored daily until 20 days postinfection. The experiments were conducted in compliance with the internationally accepted principles for laboratory animal use and care as contained in the Canadian Council on Animal Care guidelines on animal use protocol review.

## RESULTS

### Isolated compounds and their structures.

**FIG 1:**
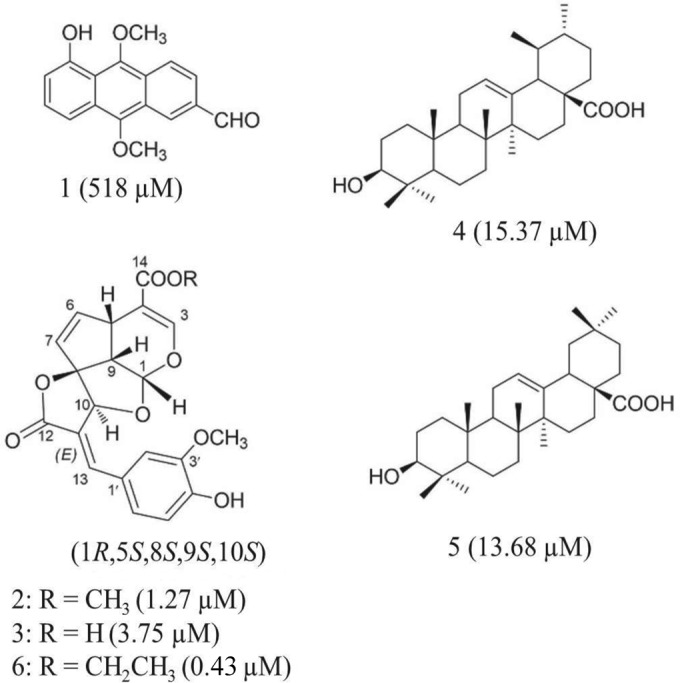
Chemical structures and activities of compounds isolated from M. lucida. Structures of novel tetracyclic spirolactone iridoids, ML-2-2, moludicin (compound 2), ML-2-3 (compound 3), and ML-F52 (compound 6), and known compounds, oruwalol (compound 1), ursolic acid (compound 4), and oleanolic acid (compound 5), isolated from Morinda lucida leaves with their respective IC_50_s after 48 h of incubation.

Bioassay-guided column chromatography resulted in the isolation of six compounds, including three novel compounds (molucidin, ML-2-3, and ML-F52). The rest are oruwalol (39) (compound 1), ursolic acid (compound 4), and oleanolic acid (compound 5) (40) (Fig. 1). The three novel compounds (molucidin, ML-2-3, and ML-F52) were found to share a unique tetracyclic iridoid skeleton. Chemical characteristics of the respective compounds are as follows.

### Molucidin.

Colorless crystal; [α]^25^_D_-188.5° (*c* 1.0, CHCl_3_); HR-ESI-MS *m/z*, 399.1084 (M + H)^+^ (calculated for C_21_H_19_O_8_, 399.1080); ^1^H-NMR (CDCl_3_, 400 MHz) δ, 3.58 (1H, dd, *J* = 10.0, 6.0 Hz, H-9), 3.78 (3H, s, 14-COOCH_3_), 3.96 (3H, s, 3′-OCH3), 4.05 (1H, dt, *J* = 10.0, 2.0 Hz, H-5), 5.22 (1H, s, H-10), 5.63 (1H, dd, *J* = 6.4, 2.4 Hz, H-7), 5.64 (1H, d, *J* = 5.6, H-1), 6.03 (1H, dd, *J* = 6.4, 2.0 Hz, H-6), 6.99 (1H, d, *J* = 8.0 Hz, H-5′), 7.26 (1H, dd, *J* = 8.0, 2.0 Hz, H-6′), 7.43 (1H, d, *J* = 2.0 Hz, H-2′), 7.46 (1H, s, H-3), 7.78 (1H, s, H-13); and^13^C-NMR (CDCl_3_, 100 MHz) δ, 102.4 (C-1), 153.0 (C-3), 109.6 (C-4), 38.5 (C-5), 141.1 (C-6), 125.9 (C-7), 104.4 (C-8), 54.3 (C-9), 82.2 (C-10), 120.1 (C-11), 170.0 (C-12), 144.9 (C-13), 166.7 (C-14), 51.7 (14-COOCH_3_), 126.5 (C-1′), 112.4 (C-2′), 149.1 (C-3′), 147.0 (C-4′), 115.1 (C-5′), 125.9 (C-6′), 56.0 (3′-OCH_3_). Molucidin has been described in our previous study (29).

### Compound 3 (ML-2-3).

Colorless crystal; [α]^25^_D_-89.2° (*c* 0.35, CHCl_3_); HR-ESI-MS *m/z*, 385.0925 (M + H)^+^ (calculated for C_20_H_17_O_8_, 385.0923); ^1^H-NMR (CDCl_3_,400 MHz) δ, 3.60 (1H, dd, *J* = 10.0, 6.0 Hz, H-9), 3.95 (3H, s, 3′-OCH3), 4.05 (1H, dt, *J* = 10.0, 2.0 Hz, H-5), 5.28 (1H, s, H-10), 5.67 (1H, dd, *J* = 6.4, 2.4 Hz, H-7), 5.68 (1H, d, *J* = 5.6, H-1), 6.06 (1H, dd, *J* = 6.4, 2.0 Hz, H-6), 6.92 (1H, d, *J* = 8.0 Hz, H-5′), 7.25 (1H, dd, *J* = 8.0, 2.0 Hz, H-6′), 7.49 (1H, d, *J* = 2.0 Hz, H-2′), 7.50 (1H, s, H-3), 7.75 (1H, s, H-13); and ^13^C-NMR (CDCl_3_, 100 MHz) δ, 103.6 (C-1), 153.9 (C-3), 110.2 (C-4), 39.2 (C-5), 141.9 (C-6), 126.9 (C-7), 105.7 (C-8), 54.9 (C-9), 83.0 (C-10), 120.0 (C-11), 169.2 (C-12), 145.9 (C-13), 171.7 (C-14), 127.2 (C-1′), 113.7 (C-2′), 151.1 (C-3′), 148.8 (C-4′), 116.2 (C-5′), 126.0 (C-6′), 56.1 (3′-OCH_3_).

The molecular formula of ML-2-3 (compound 3) was defined as C_20_H_17_O_8_ on the basis of the HR-ESI-MS experiment. The ^1^H and ^13^C-NMR spectra of ML-2-3 showed two relatively downfield CH signals at δ 103.6 (C-1) and δ 153.9 (C-3) correlated with H-1 at δ 5.68 (d, *J* = 5.6 Hz) and H-3 at δ 7.49 (br s) in the heteronuclear multiple-quantum correlation (HMQC) spectrum together with a quaternary carbon at δ 110.2 (C-4) suggested an iridoid-like structure (41). In addition, the presence of a 1,3,4-trisubstituted aromatic ring with a typical ABX coupling pattern (δ 7.49 [d, *J* = 2.0, H-2′], 6.92 [d, *J* = 8.0 Hz, H-5′], and 7.25 [dd, *J* = 8.0, 2.0 Hz, H-6′]) in the ^1^H NMR spectrum, a carbonyl carbon at δ 169.2 (C-12), and two olefinic carbons at δ 120.0 (C-11) and 145.9 (C-13) proposed a coumaroyl-like (C_6_-C_3_) moiety, which links to the iridoid nucleus (30). Furthermore, along with a downfield quaternary carbon at δ 105.7 (C-8) and a CH group (δ 83.0 [C-10] and 5.28 [br s, H-10]), the heteronuclear multiple-bond correlation (HMBC) spectrum revealed the key correlations of H-1/C-10, H-10/C-12, H-10/C-13, and H-13/C-10, indicating the connection of the C_6_-C_3_ moiety with the iridoid nucleus to form a rigid spirolactone tetracyclic ring skeleton similar to plumericin (42), oruwacin (43), and prismatomerin (44).

### Compound 6 (ML-F52).

White amorphous powder; [α]^25^_D_-62° (*c* 0.33, CHCl_3_); HR-ESI-MS *m/z*, 413.1249 (M + H)^+^ (calculated for C_22_H_21_O_8_, 413.1236); ^1^H-NMR (CDCl_3_, 400 MHz) δ, 1.31 (3H, t, *J* = 7.2 Hz, -OCH_2_CH_3_), 3.56 (1H, dd, *J* = 9.6, 6.0 Hz, H-9), 3.96 (3H, s, 3′-OCH3), 4.06 (1H, dt, *J* = 9.6, 2.0 Hz, H-5), 4.24 (2H, q, *J* = 3.6 Hz, -OCH_2_CH_3_), 5.22 (1H, s, H-10), 5.63 (1H, dd, *J* = 6.4, 2.4 Hz, H-7), 5.64 (1H, d, *J* = 5.6, H-1), 6.03 (1H, dd, *J* = 6.4, 2.0 Hz, H-6), 7.00 (1H, d, *J* = 8.0 Hz, H-5′), 7.25 (1H, dd, *J* = 8.0, 2.0 Hz, H-6′), 7.43 (1H, d, *J* = 2.0 Hz, H-2′), 7.46 (1H, s, H-3), 7.77 (1H, s, H-13); and ^13^C-NMR (CDCl_3_, 100 MHz) δ, 102.3 (C-1), 152.7 (C-3), 109.8 (C-4), 38.5 (C-5), 141.1 (C-6), 126.4 (C-7), 104.4 (C-8), 54.3 (C-9), 82.2 (C-10), 120.2 (C-11), 170.0 (C-12), 144.8 (C-13), 166.3 (C-14), 60.5 (-OCH_2_CH_3_), 14.3 (-OCH_2_CH_3_), 126.4 (C-1′), 112.4 (C-2′), 149.1 (C-3′), 147.0 (C-4′), 115.1 (C-5′), 126.0 (C-6′), 56.0 (3′-OCH_3_). The structure of ML-F52 including stereochemistry was assigned by means of the NMR spectra and optical rotation value.

### Comparison of the compounds.

NMR data of molucidin were very similar to those of ML-2-3 (see below). The presence of a methyl group, however, was evident from ^13^C NMR signal at δ 51.7 (OCH_3_) and ^1^H NMR signal at δ 3.78 (s, OCH_3_) and the HR-ESI-MS showing a molecular ion peak at *m/z* 399.1084 (M + H)^+^ (calculated for C_21_H_19_O_8_, 399.1080).

NMR data of ML-2-3 were found to have close similarity with those reported for prismatomerin except for the 1,3,4-trisubstituted aromatic ring as above and the free carboxylic function at C-14 of ML-2-3 featured by a relatively downfield shifted signal at δ 171.4.

The relative configuration of ML-2-3 elucidated by the NOESY experiment is as follows. The NOESY spectrum of ML-2-3 revealed the cross-peaks of H-1/H-9 and H-5/H-9 indicating H-1, H-5, and H-9 are cofacially oriented. Furthermore, the NOESY correlations of H-10 at δ 5.28 with H-2′ at δ 7.47 and H-6′ at δ 7.25 and no observed NOESY interaction of H-10 with H-13 supported *E*-configuration of the C-11=C-13 double bond in ML-2-3. Based on these findings, the relative configuration of ML-2-3 was determined to be similar to that of prismatomerin (44). Recently, the absolute configuration of the spirolactone tetracyclic iridoids, including plumericin, oruwacin, and prismatomerin has been well assigned by the combination of NMR spectra and optical rotation using computational calculation and experimental value (45). Subsequently, the relative configuration of ML-2-3 defined the absolute configuration of its rigid spirolactone tetracyclic skeleton as of either (1*R*,5*S*,8*S*,9*S*,10*S*) or (1*S*,5*R*,8*R*,9*R*,10*R*), and on the basis of the negative optical rotation value ([α]^25^_D_-89.2° [*c* 0.35, CHCl_3_]), the absolute configuration of ML-2-3 was then assigned as (1*R*,5*S*,8*S*,9*S*,10*S*).

The ^1^H and ^13^C NMR spectra of ML-F52 (compound 6) closely resembled the data for molucidin apart from the appearance of signals arising from an ethyl moiety (δ 4.24 [2H, q, *J* = 3.6 Hz, -OCH_2_CH_3_], 1.31 [3H, t, *J* = 7.2 Hz, -OCH_2_CH_3_]; δ 60.5 [-OCH_2_CH_3_], 14.3 [-OCH_2_CH_3_]) instead of the methyl group in molucidin. This finding was further evident by the high-resolution mass spectrometry (HRMS) result of a quasimolecular ion peak at *m/z* 413.1249 (M + H)^+^ (calculated for C_22_H_21_O_8_, 413.1236). The linkage of the ethyl group to C-14 was confirmed by an HMBC correlation between the methylene signal at δ 4.24 (-OCH_2_CH_3_) and C-14 at δ 166.3.

### Antitrypanosomal activities and cytotoxicity of isolated compounds.

The three novel compounds, molucidin (compound 2), ML-2-3 (compound 3), and ML-F52 (compound 6) had antitrypanosomal activities with IC_50_s of 1.27 μM, 3.75 μM, and 0.43 μM, respectively. Two known compounds, ursolic acid (compound 4) and oleanolic acid (compound 5) had moderate activities with IC_50_s of 15.37 μM and 13.68 μM, respectively. Oruwalol (compound 1) had no significant activity with a 518 μM IC_50_ (Fig. 1).

Cytotoxicity assay results (Table 1) showed molucidin and ML-F52 to have relatively high toxicity with IC_50_s between 4.74 μM and 14.24 μM against all cell lines tested. On the other hand, ML-2-3 did not show any cytotoxicity with ≤50 μM among all cell lines. Regarding the selectivity index (SI) values, which represent how the compounds inhibit the growth of the target organisms, ML-2-3 and ML-F52 but not molucidin were >10 for all of the cell lines, suggesting that ML-2-3 and ML-F52 might be ideal lead compounds compared with molucidin for antitrypanosomal activity.

**TABLE 1:** Antitrypanosomal activities and cytotoxicities of three novel tetracyclic iridoids, molucidin, ML-2-3, and ML-F52, against four types of human fibroblast cell lines^*a*^

Cell type	Cell line	IC_50_ (μM), 48 h			SI		
		Molucidin (1.27)	ML-2-3 (3.75)	ML-F52 (0.43)	Molucidin	ML-2-3	ML-F52
Normal skin fibroblast	NB1RGB	7.11	>50	4.74	5.60	>13.33	11.02
Normal lung fibroblast	HF-19	14.24	>50	10.94	11.21	>13.33	25.44
Normal lung	Hs888Lu	9.29	>50	8.89	7.31	>13.33	20.67
Normal liver	Chang liver	9.34	>50	18.13	7.35	>13.33	42.16

a SI values were obtained with values of antitrypanosomal activities and cytotoxicity on each compound, molucidin (compound 2), ML-2-3 (compound 3), and ML-F52 (compound 6).

### Mechanisms of trypanocidal activities for molucidin, ML-2-3, and ML-F52.

**FIG 2:**
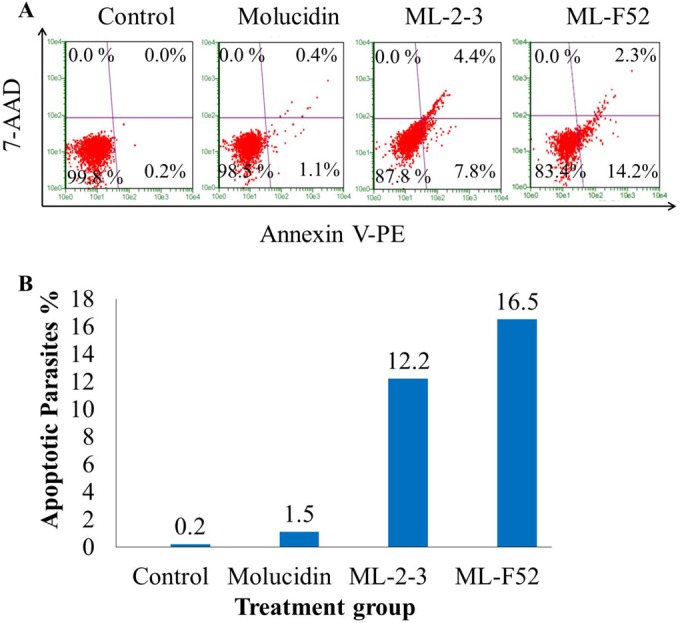
ML-2-3- and ML-F52-induced apoptotic cell death in Trypanosoma parasite cells. (A) Trypanosoma parasites incubated with various concentrations of compounds ranging from 0 μM to 50 μM for 24 h at 37°C and 5% CO_2_ were subjected to a Nexin assay. Dot plots were generated by flow cytometry. Control represents negative control (untreated population). ML-2-3- and ML-F52-induced strong apoptosis at minimum concentrations of 6.25 μM and 0.78 μM, respectively, whereas molucidin showed no significant apoptotic induction. (B) Percentages of apoptotic parasites within the different compound-treated populations.

Recently, apoptosis-like death mechanisms in trypanosomatid parasites were found (46–48), which may actually be exploited as a possible target to fight against trypanosomiasis. To investigate if the novel compounds, molucidin, ML-2-3 and ML-F52, involve apoptosis-like cell death machinery in their antitrypanosomal activities, we performed a FACS Nexin assay using trypanosome parasites treated with each compound for 24 h. ML-2-3-treated Trypanosoma parasites showed significant apoptosis induction with 7.8% of early- and 4.4% of late-stage apoptotic cells compared with untreated Trypanosoma parasites with 0.2% of early- and 0% of late-stage apoptotic cells (Fig. 2). On the other hand, even five times the IC_50_ concentration of molucidin (6.25 μM) showed no significant induction of apoptosis, 0% of late-stage apoptotic cells, and 1.1% of early-stage apoptotic cells (Fig. 2). ML-F52 showed the strongest induction of apoptosis with 14.2% of early- and 2.3% of late-stage apoptotic cells at a very low concentration of 0.78 μM (Fig. 2). These findings demonstrated that ML-2-3 and ML-F52 but not molucidin had apoptosis induction activity against Trypanosoma parasites (Fig. 2).

### Effects of compounds on parasite morphology and flagellum function.

**FIG 3:**
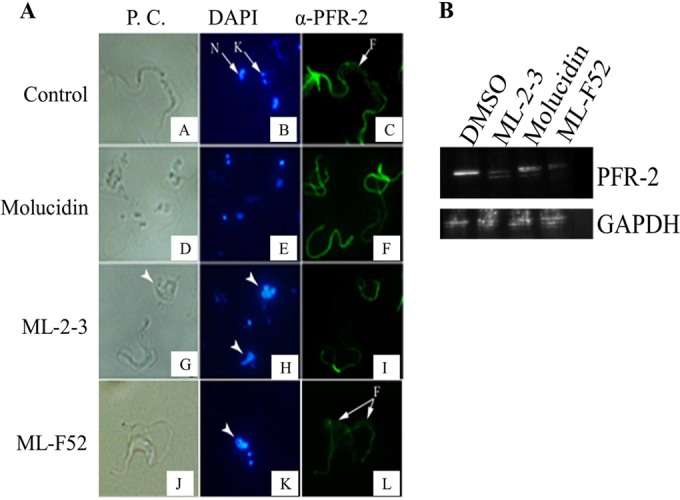
The effect of three novel compounds on parasite morphology and flagellum formation. (A) Immunohistochemistry results of Trypanosoma parasites incubated in the presence or absence of either 5 mM (4× that of the IC_50_) of molucidin, 15 mM (4× that of the IC_50_) of ML-2-3, or 0.43 mM (IC_50_) of ML-F52. DAPI stained both nucleus (N) and kinetoplast (K) in parasite cells. Parasite flagellum was stained by fluorescein isothiocyanate (FITC) (green) using the anti-PFR-2 antibody. ML-2-3 and ML-F52 induced fragmented distorted nuclei, which are indicated by arrowheads (H and K), but molucidin did not (E). The expression of PFR-2 was suppressed by ML-2-3 and ML-F52 (I and L). In addition, ML-2-3 induced round shaped cells having shortened flagella. (G) ML-F52 induced parasites that have two set of kinetoplasts and two set of flagella in one parasite (K and L). (B) The quantification analysis of the PFR-2 protein in trypanosoma cells incubated with molucidin, ML-2-3, or ML-F52 (concentrations are the same as the immunohistochemistry study) was done by Western blotting using the anti-PFR-2 antibody. ML-2-3 and ML-F52 but not molucidin suppressed the expression of PFR-2 proteins. Glyceraldehyde 3-phosphate dehydrogenase (GAPDH) proteins were detected as an internal control.

PFR-2, which is expressed in the paraflagellar rod, plays a key role not only in motility but also in cell cycle and proliferation. PFR-2 knockout in trypanosome parasites caused incomplete cell division and resulted in aggregation of parasites (34, 35). We also found a lot of aggregated parasites (data not shown). We therefore investigated the involvement of PFR-2 as a possible target candidate for the novel tetracyclic iridoids by immunohistochemistry using anti-PFR-2 antibody as well as DAPI, which stains parasite nucleus and kinetoplast. We observed intact kinetoplast but totally disintegrated nuclei in stumpy-like parasites in the molucidin-treated group (Fig. 3A, D to F). Flagella, however, appeared to be normal with significant expression of PFR-2 (Fig. 3A, D to F). ML-2-3 and ML-F52 induced fragmented nuclei with normal kinetoplast. ML-2-3 induced a typical short stumpy form while ML-F52 caused abnormal cells that had two sets of kinetoplasts and flagellum with fragmented nuclei (Fig. A, K and L). ML-2-3- and ML-F2-treated cells appeared to have less expression of PFR-2 proteins in their flagella (Fig. 3A, G to L). We further performed quantitative Western blotting using an anti-PFR-2 antibody against parasites treated with molucidin, ML-2-3, and ML-F52. The quantification of the PFR-2 protein clearly showed the suppression of PFR-2 expression by ML-2-3 and ML-F52 but not by molucidin (Fig. 3B). Interestingly, in the ML-F52-treated group, we found a large population of parasites having two sets of kinetoplasts and two sets of flagella in a cell.

### PFR-2 suppression and cell cycle alteration preceded apoptosis induction in ML-2-3-treated parasites.

**FIG 4:**
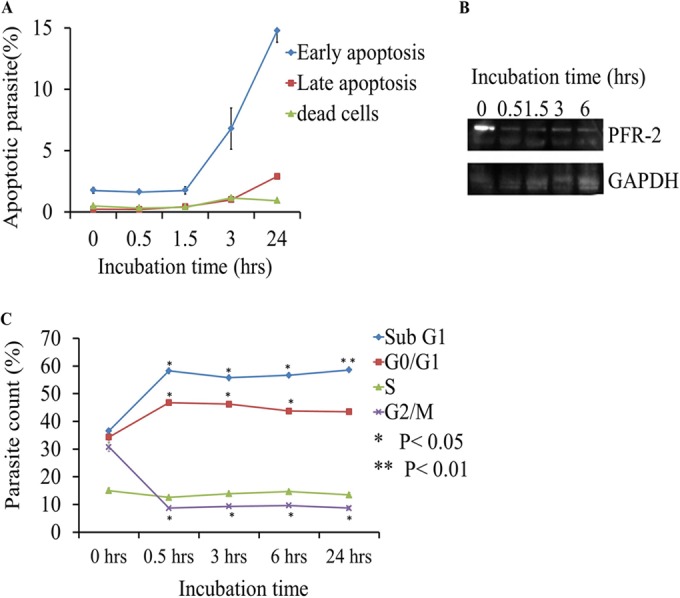
PFR-2 suppression and cell cycle alteration preceded an apoptosis event in ML-2-3-treated Trypanosoma parasites. (A) Time course Nexin apoptosis assay using Trypanosoma cells treated with 15 μM ML-2-3. Trypanosoma parasites were cultured in the presence of 15 μM ML-2-3 for different time periods (0, 0.5, 1.5, 3, and 24 h), and then percentages of apoptotic and dead parasites were obtained using flow cytometry. The values are represented as the means from three different experiments. (B) Time course Western blot analysis on PFR-2 suppression using Trypanosoma cells treated with 15 μM ML-2-3. GAPDH was used as a loading control. (C) Time course cell cycle analysis using the same conditions as those for the time course Western blot analysis with ML-2-3-treated Trypanosoma cells. Percentages of each phase of cells during cell cycles are shown as a line graph for up to 24 h of incubation.

We demonstrated that ML-2-3 and ML-F52 involved apoptosis-like cell death in their growth suppression. Moreover, we found these two compounds inhibited PFR-2 expression in parasite flagellum. The flagellum is also known to have a significant role in cell cycle and growth, and PFR-2 is one of the responsible proteins for those activities. These findings led us to the hypothesis that PFR-2 may be a possible target of ML-2-3 and ML-F52, resulting in cell cycle alteration and apoptotic cell death. We, therefore, investigated the timing of all events, including apoptosis and PFR-2 suppression as well as cell cycle alteration. The time course analysis from 0.5 h to 24 h of ML-2-3-treated parasites was performed using the Nexin assay, Western blotting with PFR-2 antibody, and the FACS cell cycle assay. ML-2-3-treated trypanosome parasites showed that induction of early and late stages of apoptosis occurred at 3 h and continued to 24 h of exposure (Fig. 4A). However, Western blotting showed that PFR-2 protein expression was significantly suppressed within 0.5 h of parasite exposure to ML-2-3 (Fig. 4B). Significant changes in the parasites' cell cycle were also found to be induced within 0.5 h of parasite exposure to ML-2-3, in which subG_1_ and G_0_/G_1_-phase cells increased from 37% to 60% and 35% to 46%, respectively; whereas G_2_/M-phase cells decreased from 31% to 9% (Fig. 4C). These changes, however, continued through to 24 h. S-phase cells stayed stable. These results, therefore, suggested that suppression of PFR-2 in flagellum and alteration in the G_0_/G_1_ phase of cell cycle preceded induction of apoptosis in ML-2-3-treated parasite cells.

### Evaluation of mouse *in vivo* efficacy for molucidin, ML-2-3, and ML-F52.

**FIG 5:**
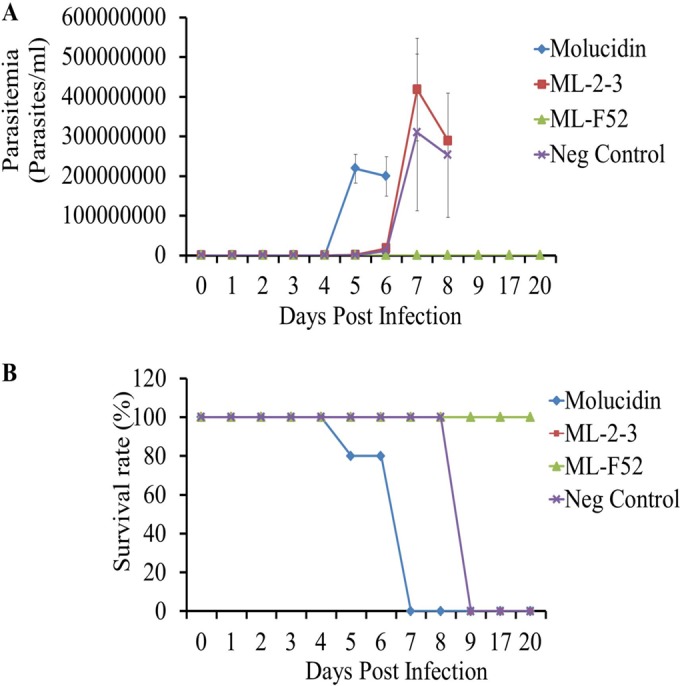
*In vivo* efficacy test of three novel compounds, molucidin, ML-2-3, and ML-F52. (A) Parasitemia changes for 20 days postinfection. Five consecutive daily shots of 30 mg/kg of each compound were inoculated (intraperitoneally) as well as vehicle-treated mice (Neg Control). ML-F52-treated mice showed no parasitemia for 20 days. (B) Survival rate curve for 20 days postinfection. ML-F52-treated group showed 100% cure for 20 days postinfection.

Molucidin, ML-2-3, and ML-F52 were evaluated for *in vivo* efficacy using a mouse model. Six-week-old female BALB/c mice (average of 20 g body weight) (*n* = 5 per group) infected with 1 × 10^3^ cells of T. brucei (TC-221 strain) were administered intraperitoneally 30 mg/kg of each compound 6 h postinfection and continued daily afterwards for 5 consecutive days. Results showed that 30 mg/kg of ML-F52 completely cleared trypanosome parasites and ensured the survival of mice for 20 days postinfection, while vehicle control mice died at day 9. ML-2-3-treated mice also died at 9 days postinfection. Molucidin-treated mice were all dead by 7 days postinfection (Fig. 5).

## DISCUSSION

The main aim of this study was to identify antitrypanosomal compounds from the extracts of M. lucida leaves, which are popularly used as a traditional medicine to treat parasitic diseases in West Africa. Several groups had already reported that the leaves of Morinda lucida possessed antitrypanosomal properties (15, 28, 49); however, the responsible active components were yet to be isolated. Hence, novel compounds with trypanosomal activity isolated from this plant might be good candidates for new chemotherapy for sleeping sickness in humans and Nagana in animals. We recently published one of the novel tetracyclic iridoids, ML-2-2 as molucidin which is an enantiomer of oruwacin (29).

In this study, two more active novel compounds, ML-2-3 and ML-F52, with three other known compounds; oruwalol (compound 1), ursolic acid (compound 4) (30), and oleanolic acid (compound 5) were identified together with molucidin from the extract of M. lucida leaves (Fig. 1). Molucidin, ML-2-3, and ML-F52 have novel tetracyclic iridoid skeletons, and their absolute configurations were determined (1*R*,5*S*,8*S*,9*S*,10*S*). The chemical structures revealed that their side chains have different functional groups at C-4. ML-2-3 has a carboxylic acid while molucidin and ML-F52 have methyl and ethyl ester functional groups, respectively (Fig. 1).

Molucidin, ML-2-3, and ML-F52 (Fig. 1) had significant trypanocidal activities, with IC_50_s of 1.27 μM, 3.75 μM, and 0.43 μM, respectively. Cytotoxicity assays showed molucidin to be more toxic than ML-2-3 and ML-F52 in all of the normal fibroblast cells tested (Table 1). SI values of three novel compounds demonstrated ML-2-3 and ML-F52 to be more specific against trypanosome parasites than molucidin.

We also demonstrated that ML-2-3 and ML-F52 induced apoptosis in Trypanosoma cells (Fig. 2). This finding was supported by two other observations that ML-2-3 and ML-F52 caused fragmented nuclei in DAPI-stained parasite cells (Fig. 3A) and an increase of the sub-G_1_-phase population in cell cycle analysis with ML-2-3-treated cells (Fig. 4C). Moreover, ML-2-3 and ML-F52 suppressed the expression of PFR-2 (Fig. 3B). It is known that flagella play a key role not only in motility but also cell cycle progression and cell division (29, 30, 47). Indeed, we also demonstrated that cell cycle alteration occurred in ML-2-3-treated cells as well as PFR-2 suppression within 0.5 h of ML-2-3 treatment. Those events preceded an induction of apoptosis that was observed within 3 h of incubation. These findings, therefore, suggest that ML-2-3 and ML-F52 affect parasite flagellum formation potentially through suppression of PFR-2 expression, which may result in cell cycle disorder and eventually in death of Trypanosoma parasites by apoptosis-like death signal. A study in 2006 showed that PFR-2 knockdown induced a flagellum beat defect, which eventually caused the incompletion of cytokinesis. As a result, PFR-2 knockdown parasites had double or triple flagella (50). Interestingly, in our experiments with Trypanosoma cells, we noticed a significant increase in parasites having two flagella in ML-F52-treated cells (Fig. 3A, L).

Molucidin, on the other hand, showed neither apoptotic induction nor PFR-2 suppression capabilities in Trypanosoma cells but caused complete nuclei disintegration as shown by DAPI stain. Although three tetracyclic iridoids have activities *in vitro*, they may also have different targets and mechanisms of action. Further mechanistic studies will be necessary to establish a complete profile of actions of each compound as well as to confirm the toxicity of molucidin and explore other beneficial scientific uses of this novel compound.

A mouse *in vivo* efficacy test of molucidin, ML-2-3, and ML-F52 against T. brucei brucei parasites (Tc-221 strain) showed that 5 consecutive daily shots of 30 mg/kg ML-F52 showed complete clearance of parasitemia, resulting in a 100% cure for 20 days postinfection (Fig. 5). Molucidin, however, showed severe toxicity, which eventually caused death at day 7. These results revealed that ML-F52 might be the best lead compound for the development of new chemotherapy against trypanosome.

In addition to the antitrypanosomal activities observed, data from preliminary studies also showed anti-Plasmodium activities of molucidin, ML-2-3, and ML-F52 against Plasmodium falciparum in vitro, while molucidin also had significant efficacy against Plasmodium yoelii in preliminary *in vivo* studies using BALB/c mice (unpublished data).

Our current findings suggest that the novel tetracyclic iridoids molucidin, ML-2-3, and ML-F52 may not only be active against T. brucei parasites but other protozoan parasites as well, which therefore makes them promising lead compounds for new chemotherapies against infections caused by protozoan parasites.
